# Case report: Atypical young case of MV1 Creutzfeldt-Jakob disease with unusually long survival

**DOI:** 10.3389/fncel.2024.1518542

**Published:** 2025-01-03

**Authors:** Lucie Yeongran Ahn, Mark L. Cohen, Ignazio Cali, Tia Russell, Jessica Ludwig, Xun Jia, Alberto Bizzi, Lawrence B. Schonberger, Ryan A. Maddox, Rohini Paul, Tania C. Ghazarian, Jaspreet Garcha, Mostafa Hammoudi, Brian Stephen Appleby

**Affiliations:** ^1^Medical Scientist Training Program, Case Western Reserve University, Cleveland, OH, United States; ^2^Department of Pathology, Case Western Reserve University, School of Medicine, Cleveland, OH, United States; ^3^National Prion Disease Pathology Surveillance Center, Case Western Reserve University, School of Medicine, Cleveland, OH, United States; ^4^Neuroradiology Unit, Fondazione Istituto di Ricovero e Cura a Carattere Scientifico (IRCCS) Istituto Neurologico Carlo Besta, Milan, Italy; ^5^Centers for Disease Control and Prevention (CDC), U.S. Department of Health and Human Services (USDHHS), Atlanta, GA, United States; ^6^Department of Psychiatry, Kaiser Permanente San Jose Medical Center, Graduate Medical Education, San Jose, CA, United States; ^7^Department of Psychiatry, Loma Linda University School of Medicine, Graduate Medical Education, Loma Linda, CA, United States; ^8^Department of Internal Medicine, Community Memorial Health Systems, Ventura, CA, United States; ^9^Department of Neurology, Community Memorial Health Systems, Ventura, CA, United States; ^10^Department of Neurology, Case Western Reserve University, School of Medicine, Cleveland, OH, United States; ^11^Department of Psychiatry, Case Western Reserve University, School of Medicine, Cleveland, OH, United States

**Keywords:** sporadic CJD (sCJD), prion disease, young-onset CJD, rapidly progressing dementia, Creutzfeldt-Jakob disease (CJD)

## Abstract

Creutzfeldt-Jakob disease (CJD) is a rare, fatal, rapidly progressive neurodegenerative disease resulting from an accumulation of misfolded prion proteins (PrP). CJD affects 1–2 new individuals per million each year, and the sporadic type accounts for 90% of those cases. Though the median age at onset and disease duration vary depending on the subtype of sporadic CJD (sCJD), the disease typically affects middle-aged to elderly individuals with a median survival of 4–6 months. sCJD in younger individuals is extremely rare. Here, we present a 21-year-old female who died with a sporadic prion disease. She presented with psychiatric symptoms followed by a rapidly progressive neurocognitive and motor decline. EEG was negative for periodic sharp wave complexes; however, brain MRI was suggestive of prion disease. The cerebrospinal fluid (CSF) real-time quaking-induced conversion (RT-QuIC) assay was indeterminate. Neuropathologic examination at autopsy revealed severe neuronal loss and gliosis with secondary white matter degeneration but minimal spongiform changes and PrP deposits in the cerebellum and neocortex by immunohistochemistry. Absence of pathogenic mutations and methionine/valine heterozygosity at codon 129 of the prion protein gene (PRNP), atypical type 1 protease-resistant PrP that lacks or shows underrepresentation of the diglycosylated PrP isoform by western blot analysis, and no acquired prion disease risk factors resulted in a final diagnosis of atypical sCJD. Very young onset sCJD often has atypical clinical presentations and disease progression, neuropathological examination results, and/or laboratory test results that may confound diagnosis. It is critical to perform thorough, comprehensive evaluations to make an accurate diagnosis, which includes autopsy confirmation with histology, prion protein typing and prion gene sequencing.

## Case report

Creutzfeldt-Jakob disease (CJD) is a rare, fatal, rapidly progressive neurodegenerative disease resulting from an accumulation of misfolded prion proteins (PrP; Uttley et al., 2020; Maddox et al., 2020). Sporadic CJD (sCJD) is the most common form of CJD, comprising ~85–90% of cases (Uttley et al., 2020; Maddox et al., 2020). sCJD can be classified into subtypes based on the PrP type and polymorphism at codon 129 of the prion protein gene (*PRNP)* MM(MV)1, MV2, MM2, VV1, and VV2. sCJD typically affects middle-aged and elderly individuals with a median age at death of 67 years. Different subtypes have variable disease durations and phenotypes. MM1 and MV1 subtypes tend to present with cognitive decline with a fast progression (median: 3–4 months), while VV2 and MV2K subtypes present with cerebellar symptoms with longer disease duration (median: 6.5–17 months). MM2 and MV2C have longer disease duration with initial severe cognitive deficits (median 16 months; Collins et al., 2006). Idiopathic CJD in individuals with onset and death younger than 30 is very rare (Maddox et al., 2020). A few sCJD cases in younger individuals have been reported, with varying disease durations and phenotypes (Corato et al., 2006; Appleby et al., 2021; D'Arcy et al., 2019; Tam et al., 2023). Most young onset sCJD cases have been VV1, the rarest subtype, or sporadic fatal insomnia (sFI). It is extremely rare for a young sCJD patient to have the MV1 subtype: one case has been reported, which presented at 15-years-of-age and had a disease duration of nearly 10 years (D'Arcy et al., 2019). Herein, we describe a new case of MV1 sCJD with onset in the early 20's and a unusually long disease duration of 39 months especially for an MV1 subtype.

The patient was a 22-year-old Caucasian woman who initially presented to the emergency department with 7 month history of sudden neurocognitive decline with behavioral and personality changes. Her family history was significant only for late-onset dementia in her maternal grandmother. She had never received gonadotropin treatment, blood transfusions, or tissue transplants, and had never undergone neurosurgery. Her medical history was notable for depression and anxiety that began 3 years prior and had been stabilized with antidepressants, without any progression or additional neuropsychiatric symptoms. She had not traveled to bovine spongiform encephalopathy (BSE) affected countries during 1980–1996, nor did she have any history of venison consumption. She was not a hunter.

Her initial symptoms included new onset anxiety after she had returned home from college. Due to increased anxiety, she was seen by her primary care physician who placed the patient on scheduled escitalopram and clonazepam as needed. Despite the medication changes, her symptoms progressed.

Her anxiety continued in the following months, progressing to include symptoms of visual hallucinations, somnambulism, and insomnia. Three months after the initial onset of her symptoms, she started to demonstrate repetitive behaviors, including frequently repeating the same story and developing a ritualistic daily routine. She was insistent on washing her hands, flossing, and promptly brushing her teeth in this specific order, perseverating on this routine, and if not done in the correct order, she would forget steps. Shortly after these behavioral changes, she started to demonstrate more prominent cognitive deficits, having difficulty remembering the route to get home from her friend's house, the passcode on her phone, and even names of her immediate family members. She eventually developed difficulties in daily tasks, including putting on her shoes, often putting her left shoe on her right foot and vice versa. In addition, she demonstrated increased agitation and onset of ataxia. Previously a studious and well-adjusted college student, the patient's progressive cognitive decline and behavioral symptoms prompted her family to seek medical care.

On initial evaluation at the emergency department, the patient was noted to be avoidant and inattentive, with poor eye contact. She was alert and oriented to self and city, but not to date, including month or year. She scored a score of seven out of 30 on the Montreal Cognitive Assessment (MOCA), demonstrating deficits in short-term memory, recalling zero out of three objects, and inattentiveness, as evidenced by the inability to perform serial seven subtractions or count backward from 20.

On physical examination, she could not follow commands appropriately or perform finger-to-nose testing. Her pupils were equal, round, and reactive without neck stiffness or rigidity present. Dysarthria and aphasia were also absent. Equal and bilateral strength of upper and lower flexors and extensors were noted with +2/4 deep tendon reflexes of the biceps, brachioradialis, triceps, knee, and ankle. Unequivocal plantar reflexes were noted bilaterally with the Babinski maneuver.

**Figure 1:**
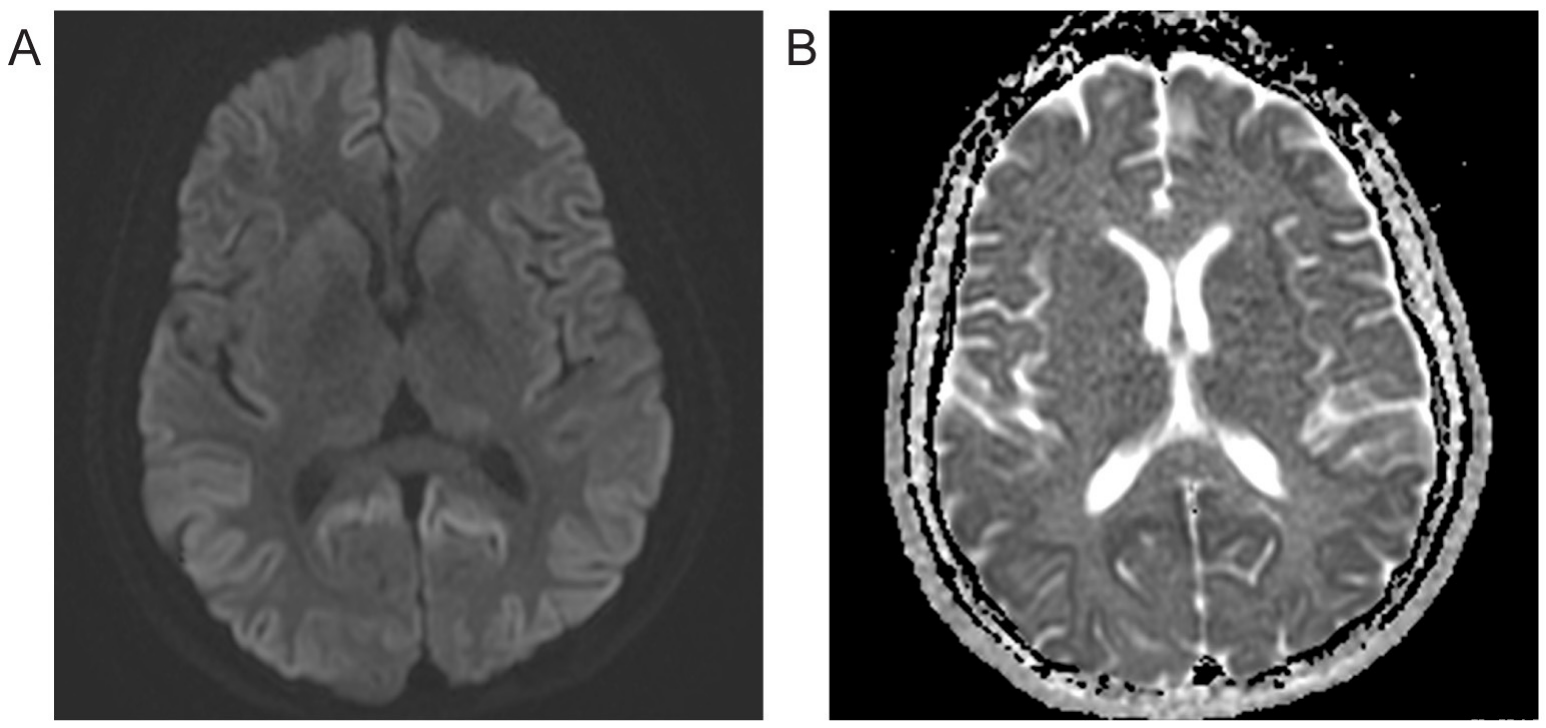
Brain MRI 8 months after symptom onset. **(A)** Diffusion-weighted imaging (DWI) reveals hyperintensity bilaterally in most cortices with additional restricted diffusion in the insula, caudate, putamen, and thalami. **(B)** Apparent diffusion coefficient (ADC) image shows hypointensity in hyperintense DWI sites, indicating restricted diffusion.

Extensive testing was performed, including an autoimmune encephalitis and rheumatologic workup, which were negative. HIV and syphilis testing were negative as well, with TSH level and liver function tests within normal limits. The sedimentation rate was mildly elevated at 21 and CRP was within normal range. EEG demonstrated a normal study in wakefulness with no periodic sharp wave complexes (PSWC) or epileptiform activity. At clinical presentation, the initial brain MRI with and without contrast imaged at the first clinical presentation revealed diffuse bilateral cerebral cortical diffusion restriction with subtle sparing of the perirolandic cortex. No associated abnormal enhancement, abnormal meningeal enhancement, or mass was noted. Brain MRI at the initial evaluation revealed restricted diffusion in parietal, frontal, and occipital cortices bilaterally, consistent with CJD (Bizzi et al., 2021). Repeat MRI several months later demonstrated additional restricted diffusion in the insula, caudate, putamen, and thalami (Figures 1A, B). The Cerebrospinal fluid (CSF) analyses conducted at the National Prion Disease Pathology Surveillance Center (NPDPSC) 8 months following the onset showed elevated CSF 14-3-3 protein, total tau level of 4,471 ng/mL, and an indeterminate real-time quaking-induced conversion (RT-QuIC) result. Sequencing detected no pathogenic mutation in *PRNP*. She was heterozygous at the non-pathogenic polymorphism located on codon 129 (M129V).

A few months after her emergency department visit, her cognition rapidly worsened with deterioration of language, executive, and visuospatial functions. She also developed delusions, headaches, severe gait ataxia with frequent falls, tremors, and hyperhidrosis, followed by myoclonus, insomnia, double incontinence, seizures, and visual dysfunction. Additionally, she developed new neuropsychiatric symptoms such as depression, anxiety, and extreme panic attacks that was no longer stabilized with antidepressants or antipsychotics. She did not undergo any life-prolonging interventions and died at home ~39 months from symptom onset (Table 1).

**Table 1:** Comparison of our case and the Canadian case from 2019.

**Characteristic**	**Our case**	**Canadian case (D'Arcy et al., 2019)**
**Patient demographic, disease course**		
Sex	Female	Female
Age at onset (years)	21	15
Disease duration (months)	39	119
**Clinical Features**		
Cognitive impairment	Yes	Yes
Visual disturbances	Yes, late	No
Ataxia	Yes	No
Myoclonus	Yes	Yes
Pyramidal symptoms	Yes	No
Psychiatric symptoms	Yes, early (depression, anxiety, panic attacks)	Yes, mild
**EEG**		
Periodic Sharp Wave Complexes (PSWC)	No	No
**Brain MRI (DWI)**		
Widespread cortical involvement	Yes	Yes
Basal ganglia involvement	Yes	Yes
**CSF laboratory findings**		
14-3-3 positive	Yes	Yes
Total tau	Yes, significantly elevated	Unknown
RT-QuIC	Indeterminate	Positive, retrospectively done.
**Neuropathological features**	Changes in cerebral cortex and cerebellum	Changes in cerebral cortex and basal ganglia.
	Abnormal (minimal) pattern of spongiosis	Minimum spongiosis.
**Genotype**	Heterozygous M129V	Heterozygous M129V
**Western blot analysis**	Lack of diglycosylated PrP; slower gel mobility than typical PrP type 1	Apparently lack of diglycosylated PrP

**Figure 2:**
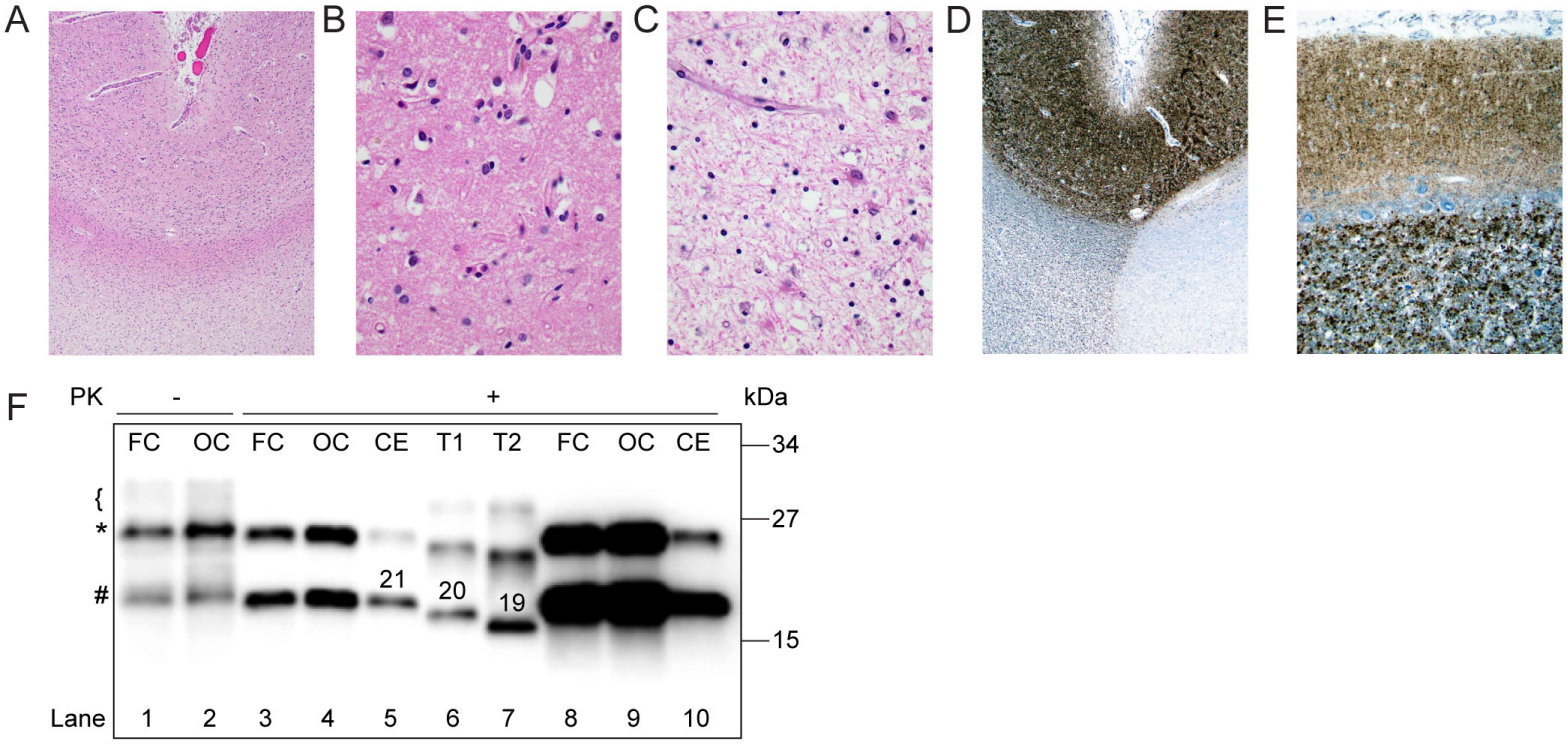
Histopathology and western blot profile of the index case. **(A)** Low-power H&E-stained section demonstrates pallor of subcortical white matter, consistent with secondary axonal degeneration (H and E x 20). **(B)** Cerebral cortical sections were remarkable for status spongiosis, rather than classical spongiform degeneration, secondary to advanced neuronal degeneration (H&E x 200). **(C)** Subcortical white matter was remarkable for severe axonal loss with reactive astrocytosis and scattered lipid-laden macrophages (H&E x 200). **(D, E)** 3 F4 immunohistochemical staining showed classical granular staining throughout the full-thickness of the cerebral cortex and cerebellar molecular layer. **(F)** Proteinase K (PK)-undigested PrP^Sc^ appears as a well-defined band of ~25 kDa (*), a less defined one of ~21 kDa (#), and a smear on the ~29–30 kDa region (bracket; lanes 1, 2). After digestion with PK, PrP^Sc^ is visualized as a doublet of ~ 25 and 21 kDa (lanes 3, 4, 8–10). Type 1 (T1, lane 6) and type 2 (T2, lane 7) controls were obtained from sCJDMM1 and -MM2, respectively. Numbers atop PrP^Sc^ bands indicate the relative molecular mass. FC, Frontal cortex; OC, occipital cortex; CE, cerebellum. Loading volume of lanes 8 and 9 is 4-fold that of lanes 3 and 4, respectively.

Neuropathologic examination revealed a pale, thin neocortex with marked neuronal loss and gliosis (“status spongiosis;” Figures 2A–C). The hippocampus was relatively spared. Classical spongiform degeneration was not discernible (Figure 2). Immunoreactive PrP deposits were detected by immunohistochemistry throughout the neocortex and cerebellum (Figures 2D, E). No intracellular PrP deposits in Purkinje cells were observed (Figure 2E). Western blot analyses showed PK-resistant PrP bands at a slightly higher molecular weight than type 1 PrP associated with classic sCJD MM1, suggesting an atypical glycoform (AG) of PrP protein (sCJDMV^AG^; Figure 2F; Tanev and Yilma, 2009; Zanusso et al., 2007; Galeno et al., 2017).

## Discussion

Very young decedents (before the age of 30) with CJD are extremely rare, and most are attributable to exogenous factors or inherited genetic mutation. Only a handful have sCJD, and these are usually either sCJDVV1 subtypes or sporadic fatal insomnia (sFI) that typically have a younger disease onset (40–50's) and a longer disease duration than other subtypes (Maddox et al., 2020; Abu-Rumeileh et al., 2018; Brown et al., 1984; Cali et al., 2020). Until recently, young onset has not been reported in classical sCJD MV1 cases or sCJD MV1 cases with atypical histopathological phenotypes (Gelpi et al., 2022; Cracco et al., 2023). The first very young-onset sCJD MV1 subtype with an unexpectedly long disease duration (119 months, which may be in part due to life-prolonging interventions) was recently reported in Canada (D'Arcy et al., 2019). Our patient, who developed her first symptoms of depression and anxiety at 21 years of age, is the second sCJDMV1 case, to our knowledge, with a notably young disease onset. The longer duration of illness seen in both cases may be attributable to young age since younger patients are less likely to have premorbid neurological conditions and comorbidities. Reported young onset sCJDs (before the age of 50) patients have, on average 1.45 months longer disease duration (Corato et al., 2006). However, in the NPDPSC's cohort of 168 sCJDMV1 cases, the mean age (67 years) of the patients with disease duration ranging 1–10 months (*N* = 137; median: 3 months) does not differ from that (68 years) of cases with a duration ranging 11–41 months (*N* = 31; median: 16 months). These data suggest that the longer survival seen in typical sCJDMV1 cases is not simply due to the young age of onset.

Classically, the median age for the sCJD MV1 subtype is 65.5 years, with a median disease duration of 5 months (Collins et al., 2006). sCJD MV1 subtype patients most commonly present with cognitive impairment such as memory loss, confusion, or disorientation (Collins et al., 2006; Gelpi et al., 2022). Certain atypical characteristics such as neuropsychiatric symptoms, headaches, and sleep disturbances are more commonly reported in young onset sCJDs (Corato et al., 2006; Appleby et al., 2007). Both the Canadian case and our case presented like most young sCJD patients who initially present with neuropsychiatric symptoms and then develop cognitive symptoms, culminating in gross neurological deficits (D'Arcy et al., 2019; Cracco et al., 2023).

Furthermore, our case characterized by underrepresentation of the diglycosylated PrP isoform (Figure 2F; D'Arcy et al., 2019). Despite the young age, the lack or marked underrepresentation of PK-resistant diglycosylated PrP argue against the diagnosis of variant CJD (Diack et al., 2019). Our patient initially presented with neuropsychiatric symptoms like depression and anxiety followed by signs of diffuse cognitive impairments such as memory loss, delusions, and headaches. Her neuropsychiatric symptoms early during her disease course could either be idiopathic or an early presentation of sCJDs (Corato et al., 2006; Appleby et al., 2007). However, given that the patient's mood was previously well-controlled with antidepressants and that she developed new and progressively worsening symptoms of depression, anxiety, and panic attacks, we attribute the latter symptoms to her new onset sCJD. The patient progressively developed worsening diffuse cognitive impairment, motor symptoms, and myoclonus. Despite the differences in the survival time between the Canadian case (119 months) and our case (39 months), the clinical progression of the two cases were similar (Table 1).

PSWC are seen in most sCJD MV1 subtype patients (Parchi et al., 1999); however, this finding is often time-dependent and may be missed in individual cases. Interestingly, neither the Canadian nor our case had the characteristic PSWCs (D'Arcy et al., 2019). Both were positive for the cerebrospinal fluid (CSF) level of the 14-3-3 protein, a surrogate marker of CJD (Castellani et al., 2004; Otto et al., 2002). RT-QuIC is a relatively new diagnostic method with high sensitivity and specificity that utilizes the PrP protein's self-replicating ability and aggregate formation (Green, 2019; Rhoads et al., 2020). The Canadian case tested positive for RT-QuIC, but our case was deemed indeterminate due to the assay's low amplitude that did not reach positivity threshold. In our case, a long lag time was also noted on CSF RT-QuIC. Early MRI images from both cases revealed hyperintensity bilaterally in most cortices, consistent with characteristic MV1 subtype images (Gelpi et al., 2022). Later, MRI images showed additional diffusion in the striatum in both cases (Figure 1).

Neuropathological examination of sCJD MV1 subtype patients reveals spongiform changes in most layers of the cortex, sparing the first layer, astrogliosis, and neuronal loss. The molecular layer is primarily affected in the cerebellum, and the hippocampus is usually spared (Parchi et al., 1999). Neuropathologic analyses of our case showed generalized cortical atrophy and signs of astrogliosis but had minimal spongiform changes. The Canadian case also had minimal spongiform changes. Immunohistochemical staining revealed a dense deposition of PrP proteins in the neocortex and cerebellum, confirming the diagnosis (Figures 2A–E). Interestingly, the western immunoblot analysis for protease-resistant PrP showed PK-resistant PrP bands at slightly higher molecular weight than type 1 PrP, suggesting an atypical glycoform of PrP protein (Figure 2F).

## Conclusion

In summary, very young sCJD patients with long disease duration may present quite differently than typical sCJD patients. To our knowledge, our case is the second reported case of a very young onset sCJD MV1 subtype with an unusually long disease duration and PrP type. Our case's MRI findings are consistent with what we would expect from an MV1 subtype patient. However, the clinical presentation, disease course, the absence of PSWCs in EEG, and indeterminate RT-QuIC results is not characteristic of sCJD MV1. Ultimately, the neuropathological exam analyses performed at the NPDPSC combined with no known risk factors for acquired prion disease provided confidence that this young patient had sporadic prion disease. An extremely rare, very young onset sCJD with a long disease duration case may present differently from the characteristic clinical presentations of sCJD. Thus, it is critical to comprehensively evaluate clinical findings, but most importantly to verify the presence and type of prion disease in these younger cases though neuropathologic evaluation at autopsy, especially since acquired prion diseases historically occur in young individuals (Rhoads et al., 2020; Manix et al., 2015). Neuropathologic evaluation of young cases in the UK was crucial for identifying variant CJD in those exposed to bovine spongiform encephalopathy (Will et al., 1996). The U.S. is currently dealing with a massive spread of an animal prion disease in the cervid species called chronic wasting disease. As such, it is more important than ever to confirm the presence and type of prion disease is the US, especially in atypical cases of prion disease (Osterholm et al., 2019).

## Data Availability

The datasets presented in this article are not readily available because, the dataset is not accessible to the general public due to privacy concerns. Requests to access the datasets should be directed to Brian Stephen Appleby, bsa35@case.edu.
